# Outcomes of allogeneic stem cell transplantation in hepatosplenic T-cell lymphoma

**DOI:** 10.1038/bcj.2015.43

**Published:** 2015-06-05

**Authors:** A Rashidi, A F Cashen

**Affiliations:** 1 Section of BMT and Leukemia, Division of Oncology, Department of Medicine, Washington University School of Medicine, St Louis, MO, USA

Hepatosplenic T-cell lymphoma (HSTCL) is a rare but aggressive type of non-Hodgkin lymphoma that primarily involves the sinusoids of the liver, red pulp of the spleen and the sinuses of the bone marrow. The specific rearrangement of the expressed T-cell receptor chain genes (TCR-αβ vs γδ) determines the subtype of the disease (HSαβTCL vs HSγδTCL). HSαβTCL is the rarer subtype with fewer than 30 cases reported in the literature.^1^ HSγδTCL is classically described in patients with inflammatory bowel disease treated with azathioprine, those on chronic immunosuppression following solid organ transplantation,^2, 3^ and patients treated with TNF-α inhibitors.^4^ On the other hand, most reported cases of HSαβTCL do not seem to have an underlying immune-related condition.^1^ Hepatosplenomegaly, cytopenias and stage IVB diseases are typical. Common cytogenetic abnormalities include isochromosome 7q and trisomy 8, and neoplastic T cells are usually CD4^-^CD8^−^.^5^ HSTCL has a tendency to affect younger individuals with a median age of 20–30 years. Whereas males are significantly more commonly affected with the γδ subtype (male:female (M:F) ratio of 10:1),^3^ the αβ subtype has a slight female preponderance (F:M ratio of 1.5:1).^1^ Without allogeneic stem cell transplantation (allo-SCT), HSTCL is an almost invariably fatal disease characterized by chemorefractoriness, unremitting clinical course, and a 5-year overall survival (OS) of <10%.^1, 3^ Remissions following donor lymphocyte infusion^6, 7, 8^ and reduced immunosuppression^9^ suggest potent graft-versus-lymphoma effects. In the North American report of outcomes in HSTCL, only one of the 13 long-term survivors had not been treated with allo-SCT.^10^

Due to the rarity of the disease and the sporadic nature of the available reports, transplant outcomes in this disease are unknown. In this article, we provide a systematic review of all previously published reports of allo-SCT in HSTCL including four previously unpublished cases from our institution. We searched PubMed for all publications until 1 March 2015 using keywords ‘hepatosplenic', ‘lymphoma' and either ‘stem cell transplantation' or ‘bone marrow transplantation'.

A total of 54 eligible cases were included in analysis (Supplementary Tables S1 and S2). Reports from Europe (54%) and North America (37%) were most frequent. The disease subtype was γδ in 87% of patients. The median (range) age of patients was 34 (8–67) years, and 73% were male. The disease was stage IV in 93% of patients and B symptoms were present in 77%. Lymphadenopathy, hepatosplenomegaly and bone marrow involvement were present in 35%, 100% and 82% of patients, respectively. The median (range) extent of bone marrow involvement was 29% (3–38). The median (range) white blood cell count, hemoglobin and platelets at presentation was 5.2 (1.2–80) × 10^9^ l^−1^, 9.2 (5.2–14.4) g dl^−1^ and 59 (4–263) × 10^9^ l^−1^, respectively. Leukocytosis, leukopneia, anemia and thrombocytopenia were present in 23%, 41%, 90% and 90% of patients, respectively. Sixty-four percent of patients had a cytogenetic abnormality by conventional karyotype analysis and/or fluorescence *in situ* hybridization. Among these patients, isochromosome 7 (77%) and trisomy 8 (46%) were the most frequent. The most common immunophenotype was CD4^−^CD8^−^ (80%), followed by CD4^−^CD8^+^ (12%). The median (range) number of prior lines of therapy was 2 (1–6), and an autologous SCT was a component of prior therapies in 12% of patients. The disease status at the time of allo-SCT was complete remission in 41%, partial remission in 43% and progressive disease in 16% of patients, respectively. The latter two groups were classified as active disease in subsequent analysis. The donor was a matched sibling in 53%, matched unrelated donor in 33%, haploidentical relative in 8% and cord blood in 6% of transplants. The source of stem cells was peripheral blood in 51%, bone marrow in 44% and cord blood in 5% of patients. Conditioning was myeloablative (MA) in 70% of patients and reduced intensity in 30%. The conditioning regimen included total body irradiation in 63% of transplants. Graft-versus-host disease (GvHD) prophylaxis was calcineurin based in all patients, with the addition of post-transplant cyclophosphamide in three haploidentical transplants and anti-thymocyte globulin in two patients.

Overall, 35% of the 44 patients with known outcome relapsed, at a median of 4-months post SCT for those with known time of relapse (Figure 1). There were no relapses after 1.5-years post SCT. Relapse-free survival (RFS) and OS relative to the time of SCT were available in 44 and 42 patients, respectively. The median (standard error) RFS and OS were 18 (5) and 68 (34) months, respectively (Figure 1). The estimated 3-year RFS and OS were 42% and 56%, respectively. The cause of death was non-relapse mortality (NRM) in 68% and relapse in 32%. The estimated 3-year NRM was 34% (Figure 1). Acute and chronic GvHD occurred in 50% and 44% of patients, respectively. In univariate analysis, the only variables significantly associated with longer RFS were female gender (Hazard ratio (HR)=0.30, 95% confidence interval (CI): 0.10–0.87, *P*=0.03; Table 1, Supplementary Figure S1) and MA conditioning (HR=0.38, 95% CI: 0.15–0.97, *P*=0.04; Table 1). The cumulative incidence of relapse was similar between males and females (*P*=0.73; Supplementary Figure S1), but NRM was significantly higher among males (3-year NRM 52% vs unreached in females, *P*=0.03; Supplementary Figure S1). Accordingly, males had shorter OS (Supplementary Figure S1), although the difference did not reach statistical significance (*P*=0.11). Females and males were not significantly different in any of the studied variables including conditioning intensity (MA in 75% of females and 67% of males, *P*=0.71). The conditioning intensity was not associated with relapse, NRM or OS (*P*=0.20, 0.18 and 0.24, respectively; Table 1, Supplementary Figure S2). However, MA conditioning was associated with longer RFS (*P*=0.04; Table 1, Supplementary Figure S2). Patients with known outcomes were then classified into two groups with RFS longer (*n*=18) or shorter (*n*=26) than 1.5 years (Supplementary Table S3). The groups were not significantly different in any of the studied variables except gender. The proportion of females was significantly higher in the group with RFS >1.5 years compared with the group with RFS <1.5 years (53% vs 15%, respectively; *P*=0.02). Taken together, female gender seemed to be independently associated with longer RFS and OS, due to lower TRM. In multivariate analysis with gender and conditioning intensity as predictors of outcome, female gender remained a borderline significant predictor of longer RFS, independent of the conditioning intensity (HR=0.30, 95% CI: 0.09–1.07, *P*=0.06).

Our results from this systematic review are consistent with those from a recently published registry-based, retrospective study by the European Society for Blood and Marrow Transplantation (*n*=18).^11^ Using an exhaustive review of the literature and including four previously unreported patients from our institution, we demonstrated that as many as 40% of patients with HSTCL who undergo allo-SCT have durable RFS. Interestingly, active disease at the time of SCT did not predict poor outcomes, highlighting the potency of the graft-versus-lymphoma effect. We identified female gender as a potentially favorable prognostic factor (lower TRM and longer RFS) for patients with HSTCL who undergo allo-SCT. Whether this association is due to an independent effect of gender or a confounder not considered in our series remains to be determined. Finally, our results suggest that patients need a close follow-up in the first 1.5 years following SCT, after which relapse is rare.

## Figures and Tables

**Figure 1:**
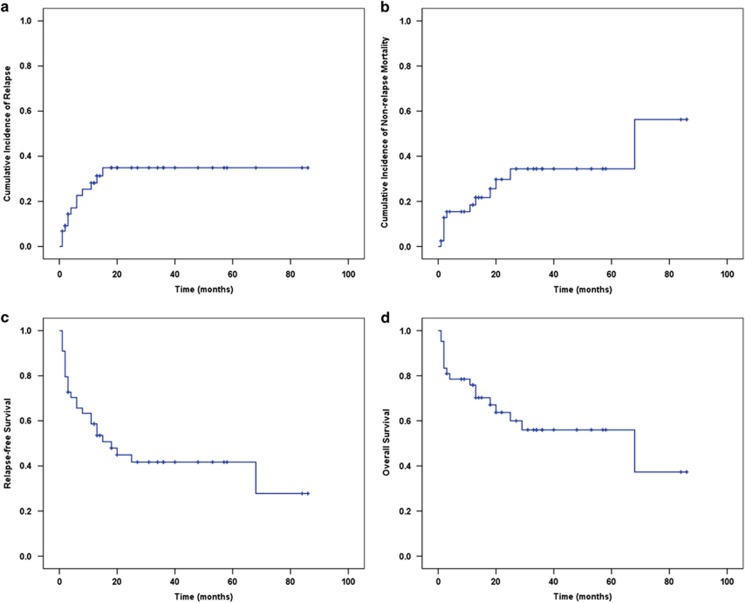
Outcomes following allo-SCT in patients with hepatosplenic T-cell lymphoma. Cumulative incidence of relapse, cumulative incidence of non-relapse mortality, RFS and OS are shown in panels **a**, **b**, **c** and **d**, respectively.

**Table 1:** Univariate survival analysis

	*RFS* a			*OS* a		
	*HR*	*95% CI*	P*-value*	*HR*	*95% CI*	P*-value*
Non-European vs European	1.39	0.62–3.09	0.42	0.39	0.11–1.35	0.14
Female vs male	0.30	0.10–0.87	0.03*	0.36	0.10–1.27	0.11
Age	1.01	0.98–1.04	0.63	1.03	0.99–1.07	0.10
B symptoms	1.37	0.46–4.08	0.57	0.76	0.24–2.42	0.64
Lymphadenopathy	0.74	0.32–1.69	0.47	1.02	0.38–2.74	0.96
White blood cell count	1.01	0.98–1.03	0.54	1.01	0.98–1.05	0.50
Hemoglobin	0.92	0.72–1.18	0.52	0.60	0.34–1.06	0.08
Platelets	0.98	0.95–1.01	0.14	0.98	0.94–1.02	0.28
γδ vs αβ	0.60	0.20–1.79	0.36	1.27	0.29–5.58	0.76
CR vs active disease^b^	0.73	0.31–1.74	0.48	0.56	0.18–1.79	0.33
Number of prior lines of therapy	1.11	0.80–1.53	0.54	0.85	0.55–1.32	0.47
Prior auto-SCT	0.52	0.12–2.23	0.38	0.45	0.06–3.41	0.44
Matched sibling vs others	1.10	0.40–3.04	0.85	1.22	0.39–3.85	0.73
PBSC vs BM	1.2	0.47–3.09	0.71	0.97	0.34–2.76	0.95
MA vs RI conditioning	0.38	0.15–0.97	0.04*	0.50	0.16–1.59	0.24
Acute GvHD	1.13	0.39–3.23	0.83	1.73	0.50–5.98	0.39
Chronic GvHD	0.42	0.13–1.35	0.14	0.66	0.19–2.27	0.51

Abbreviations: Auto-SCT, autologous stem cell transplantation; BM, bone marrow; CI, confidence interval; CR, complete remission; GvHD, graft-versus-host disease; HR, Hazard ratio; MA, myeloablative; PBSC, peripheral blood stem cells; OS, overall survival; RFS, relapse-free survival; RI, reduced intensity.

a Analysis was performed using the Kaplan–Meier method and log-rank test.

b Active disease was defined as partial remission or progressive disease.

*P<0.05.
