# Supplementary material for: Outcomes of allogeneic stem cell transplantation in hepatosplenic T-cell lymphoma
Source: Blood Cancer J. 2015 Jun 5;5(6):e318–. doi: 10.1038/bcj.2015.43 (PMC4648481; doi:10.1038/bcj.2015.43)
Supplement: Supplementary Figure Legends [file bcj201543x1.doc]

**Figure S1: Association of sex with relapse and survival following allogeneic stem cell transplantation in patients with hepatosplenic T-cell lymphoma.** Cumulative incidence of relapse, cumulative incidence of non-relapse mortality, relapse-free survival, and overall survival for males and females are shown separately in panels A, B, C, and D, respectively.

**Figure S2: Association of the conditioning intensity with relapse and survival following allogeneic stem cell transplantation in patients with hepatosplenic T-cell lymphoma.** Cumulative incidence of relapse, cumulative incidence of non-relapse mortality, relapse-free survival, and overall survival for patients with reduced-intensity and myeloablative conditioning are shown separately in panels A, B, C, and D, respectively.
