# Supplementary figures and images for: Outcomes of allogeneic stem cell transplantation in hepatosplenic T-cell lymphoma
Source: Blood Cancer J. 2015 Jun 5;5(6):e318–. doi: 10.1038/bcj.2015.43 (PMC4648481; doi:10.1038/bcj.2015.43)

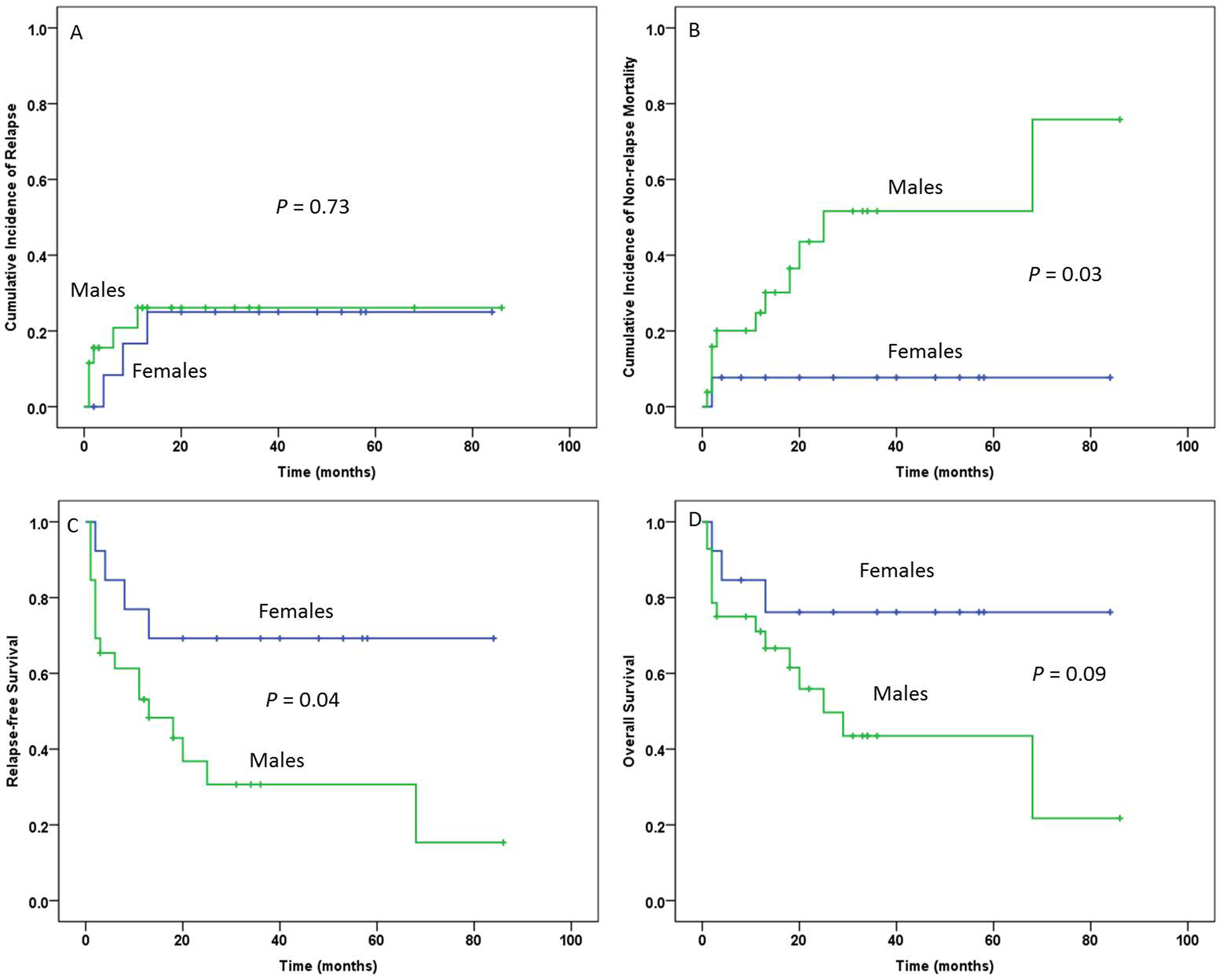

Supplement: Supplementary Figure S1 [file bcj201543x2.tif]

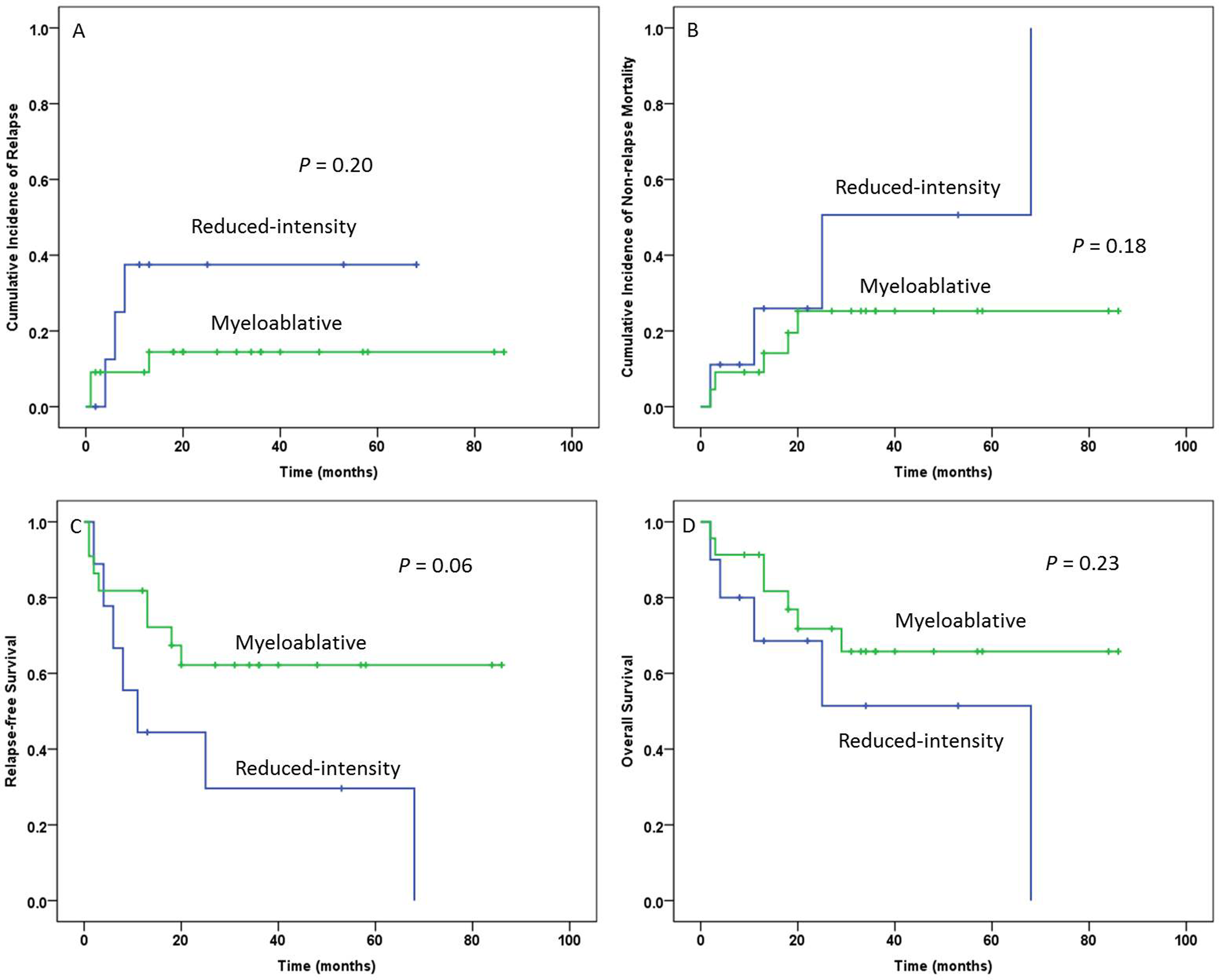

Supplement: Supplementary Figure S2 [file bcj201543x3.tif]
