# Supplementary material for: Outcomes of allogeneic stem cell transplantation in hepatosplenic T-cell lymphoma
Source: Blood Cancer J. 2015 Jun 5;5(6):e318–. doi: 10.1038/bcj.2015.43 (PMC4648481; doi:10.1038/bcj.2015.43)
Supplement: Supplementary Information [file bcj201543x4.doc]

**Supplementary Information**

**Table S1: Demographic, clinical, laboratory, and pathologic characteristics**

| **No.** | **Reference, country** | **Age/Sex** | **WBC/Hb/Plt** | **LAD/HSM** | **Type** | **Stage** | **BM** | **Cytogenetic abnormalities** | **CD4/CD8** |
| --- | --- | --- | --- | --- | --- | --- | --- | --- | --- |
| 1 | ^1^, US | 46/M | NA/L/L | -/+ | NA | IVB | - | NA | NA/NA |
| 2 | ^1^, US | 51/M | NA/L/L | -/+ | NA | IVB | + | NA | NA/NA |
| 3 | ^1^, US | 23/M | NA/L/L | -/+ | NA | IVB | - | NA | NA/NA |
| 4 | ^1^, US | 12/M | NA/L/nl | +/+ | NA | IVB | + | NA | NA/NA |
| 5 | ^1^, US | 19/M | NA/L/nl | -/+ | NA | IVA | + | NA | NA/NA |
| 6 | ^1^, US | 27/M | nl/L/L | -/+ | NA | IVB | + | NA | NA/NA |
| 7 | ^1^, US | 18/M | NA/L/L | -/+ | NA | IVB | + | NA | NA/NA |
| 8 | ^2^, US | 26/M | 49/12.4/NA | +/+ | αβ | IVB | 3% | +8 | +/- |
| 9 | ^3^, UK | 29/F | 19/13.6/263 | -/+ | αβ | IVB | + | None | -/- |
| 10 | ^4^, Japan | 26/M | 4.6/12.2/57 | -/+ | αβ | IVB | 29% | +8,i(7) | -/- |
| 11 | ^5^, Croatia | NA/M | NA/NA/L | -/+ | αβ | IVB | + | dup(7q31) | -/NA |
| 12 | ^6^, Canada | 16/M | 3/6.6/102 | -/+ | αβ | IVA | + | i(7) | -/- |
| 13 | ^7^, Italy | 48/M | 3.5/11/72 | -/+ | αβ | IVB | 15% | None | -/- |
| 14 | ^8^, Japan | 23/M | NA/NA/NA | NA/NA | γδ | IVB | NA | NA | NA/NA |
| 15 | ^9^, Japan | 25/F | 2.9/8/92 | -/+ | γδ | IVA | 13% | None | +/+ |
| 16 | ^10^, US | 8/F | NA/NA/NA | +/+ | γδ | IVB | 7% | None | -/- |
| 17 | ^11^, Italy | 34/F | NA/NA/NA | -/+ | γδ | IV | + | NA | -/- |
| 18 | ^12^, France | 19/M | 25.7/14.4/80 | -/+ | γδ | IV | + | NA | NA/+ |
| 19 | ^12^, France | 28/M | 1.2/7.4/121 | -/+ | γδ | IV | + | i(7) | -/- |
| 20 | ^12^, France | 44/M | 7/9.4/25 | -/+ | γδ | IV | + | None | -/- |
| 21 | ^13^, US | 19/M | NA/L/L | -/+ | γδ | IV | + | NA | -/+ |
| 22 | ^14^, Canada | 10/M | NA/NA/NA | NA/+ | γδ | IVB | NA | NA | NA/NA |
| 23 | ^15^, Australia | 39/M | L/L/L | -/+ | γδ | IVB | + | NA | -/- |
| 24 | ^16^, US | 46/M | nl/9/45 | -/+ | γδ | IV | + | NA | -/- |
| 25 | ^17^, US | 9/F | nl/nl/52 | -/+ | γδ | IVA | 38% | Complex, inc. +8,i(7) | -/+ |
| 26 | ^18^, Germany | 44/F | 80/9.9/32 | NA/+ | γδ | IVA | + | +8,i(7) | -/- |
| 27 | ^19^, UK | 18/M | L/L/L | -/+ | γδ | IVB | + | i(7) | -/- |
| 28 | ^20^, US | NA/NA | NA/NA/NA | NA/+ | γδ | IV | + | del(22q) | -/- |
| 29 | ^20^, US | NA/NA | NA/NA/NA | NA/+ | γδ | IV | + | Complex, inc. i(7) | -/- |
| 30 | ^21^, France | 25/M | 5.2/5.2/4 | -/+ | γδ | IV | 30% | +8,i(7) | -/- |
| 31 | ^21^, France | 21/M | 7/9.9/44 | -/+ | γδ | IV | 30% | +8,i(7) | -/- |
| 32 | ^22^, Japan | 35/M | 1.8/7.2/61 | -/+ | γδ | IVB | + | Complex | -/+ |
| 33 | ^23^, Europe | 45/M | NA/NA/NA | +/+ | γδ | IVB | + | NA | NA/NA |
| 34 | ^23^, Europe | 33/F | NA/NA/NA | NA/NA | γδ | IVA | NA | NA | NA/NA |
| 35 | ^23^, Europe | 37/F | NA/NA/NA | +/+ | γδ | IIA | - | NA | NA/NA |
| 36 | ^23^, Europe | 47/M | NA/NA/NA | +/+ | γδ | IVA | - | NA | NA/NA |
| 37 | ^23^, Europe | 36/M | NA/NA/NA | -/+ | γδ | IIIB | - | NA | NA/NA |
| 38 | ^23^, Europe | 38/F | NA/NA/NA | -/+ | γδ | IVA | + | NA | NA/NA |
| 39 | ^23^, Europe | 36/M | NA/NA/NA | +/+ | γδ | IVB | + | NA | NA/NA |
| 40 | ^23^, Europe | 25/M | NA/NA/NA | +/+ | γδ | IVB | - | NA | NA/NA |
| 41 | ^23^, Europe | 59/M | NA/NA/NA | +/+ | γδ | IVB | + | NA | NA/NA |
| 42 | ^23^, Europe | 38/F | NA/NA/NA | -/+ | γδ | IB | - | NA | NA/NA |
| 43 | ^23^, Europe | 45/M | NA/NA/NA | +/NA | γδ | IVA | + | NA | NA/NA |
| 44 | ^23^, Europe | 67/M | NA/NA/NA | +/+ | γδ | IVB | + | NA | NA/NA |
| 45 | ^23^, Europe | 39/M | NA/NA/NA | +/+ | γδ | IVB | + | NA | NA/NA |
| 46 | ^23^, Europe | 33/F | NA/NA/NA | +/+ | γδ | IVB | + | NA | NA/NA |
| 47 | ^23^, Europe | 32/M | NA/NA/NA | +/+ | γδ | IVB | + | NA | NA/NA |
| 48 | ^23^, Europe | 44/M | NA/NA/NA | +/+ | γδ | IVB | + | NA | NA/NA |
| 49 | ^23^, Europe | 59/M | NA/NA/NA | +/+ | γδ | IVB | - | NA | NA/NA |
| 50 | ^23^, Europe | 54/F | NA/NA/NA | +/+ | γδ | IVB | + | NA | NA/NA |
| 51 | Present study, US | 46/M | 1.6/7.5/76 | -/+ | γδ | IVB | 35% | None | -/- |
| 52 | Present study, US | 52/F | 9.4/6/75 | -/+ | γδ | IVB | 35% | i(7) | -/- |
| 53 | Present study, US | 31/F | 13.4/11.3/49 | -/+ | γδ | IVB | 20% | None | -/- |
| 54 | Present study, US | 42/M | 2.9/7.8/49 | -/+ | γδ | IIISB | - | None | -/- |

BM: bone marrow involvement; Hb: hemoglobin; HSM: hepatosplenomegaly; LAD: lymphadenopathy; NA: not available; OS: overall survival; Plt: platelets; UK: United Kingdom; US: United States; WBC: white blood cells

**Table S2: Treatments and outcomes**

| **No.** | **Prior lines of Tx, auto-SCT, pre-SCT status** | **Donor, BM** | **Conditioning** | | | **GvHD PPx** | **Outcome** | | | **GvHD** | |
| --- | --- | --- | --- | --- | --- | --- | --- | --- | --- | --- | --- |
|  |  |  | **Intensity** | **Flu-based** | **TBI-based** |  | **Relapse (month)** | **Death (month)** | **Death cause** | **A** | **Ch** |
| 1 | 1, -, CR | NA, NA | RI | + | - | NA | + (6) | - (51)^*^ | - | NA | NA |
| 2 | 2, -, CR | NA, NA | MA | - | + | NA | - | + (10)^*^ | NRM | NA | NA |
| 3 | 1, -, PR | NA, NA | RI | + | - | NA | + (3) | - (66)^*^ | - | NA | NA |
| 4 | 3, -, PR | NA, NA | MA | - | + | NA | + (3) | + (13)^*^ | DOD | NA | NA |
| 5 | 3, -, PR | Sib, + | MA | - | + | NA | - | - (150)^*^ | - | NA | NA |
| 6 | 2, -, CR | NA, NA | MA | - | + | NA | - | - (5)^*^ | - | NA | NA |
| 7 | 2, -, CR | NA, NA | MA | - | + | NA | - | - (3)^*^ | - | NA | NA |
| 8 | 6, -, PR | MUD, - | RI | + | - | T/MTX | + (6) | - (22) | - | - | + |
| 9 | 5, +, PR | MUD, + | MA | - | + | C/MTX | - | - (40) | - | + | + |
| 10 | 5, -, CR | Sib, - | MA | - | + | C/MTX | + (1) | - (33) | - | - | - |
| 11 | 1, -, CR | MUD, - | MA | - | + | C/MTX | - | + (13) | NRM | - | - |
| 12 | NA, NA, NA | NA, NA | NA | NA | NA | NA | + (2) | + (2) | DOD | NA | NA |
| 13 | 3, +, PD | Haplo, - | MA | + | - | T/MMF/PTCy | - | - (18) | - | - | + |
| 14 | 1, -, CR | Sib, + | MA | - | + | C/MTX | - | - (86) | - | + | + |
| 15 | 4, +, PR | Cord, - | MA | - | + | C/MTX | - | - (58) | - | + | - |
| 16 | 2, -, PR | Sib, + | MA | - | + | T/MTX | - | - (36) | - | + | - |
| 17 | 4, -, PR | NA, + | NA | NA | NA | NA | - | + (2) | NRM | NA | NA |
| 18 | 1, -, CR | NA, + | NA | NA | NA | NA | + (15) | + (25)^*^ | DOD | NA | NA |
| 19 | 1, -, CR | NA, + | NA | NA | NA | NA | - | + (6)^*^ | NRM | NA | NA |
| 20 | 1, -, PR | NA, + | NA | NA | NA | NA | - | + (9)^*^ | NRM | NA | NA |
| 21 | 2, -, PD | Sib, - | NA | NA | NA | NA | - | - (12) | - | NA | NA |
| 22 | 3, -, NA | MUD, - | MA | - | + | NA | - | - (12) | - | - | + |
| 23 | 2, -, PD | Sib, - | MA | - | + | NA | + (1) | - (9) | - | + | - |
| 24 | 1, -, PD | NA, + | NA | NA | NA | NA | - | + (1) | NRM | NA | NA |
| 25 | 1, -, NA | NA, + | NA | NA | NA | NA | NA | NA | NA | NA | NA |
| 26 | 1, -, PR | Sib, - | MA | - | + | NA | + (13) | + (13) | DOD | NA | NA |
| 27 | 4, -, PR | MUD, NA | NA | NA | NA | NA | - | + (2) | NRM | NA | NA |
| 28 | 1, -, CR | NA, NA | NA | NA | NA | NA | - | - (36)^*^ | - | NA | NA |
| 29 | 1, -, CR | NA, NA | NA | NA | NA | NA | - | - (14) | - | NA | NA |
| 30 | NA, NA, NA | NA, + | NA | NA | NA | NA | + (1) | + (1) | DOD | NA | NA |
| 31 | NA, NA, NA | NA, + | NA | NA | NA | NA | - | - (3) | - | NA | NA |
| 32 | 1, -, PR | NA, NA | NA | NA | NA | NA | + (11) | - (15) | - | NA | NA |
| 33 | 1, -, PR | Sib, - | MA | + | - | C/MMF/ATG | - | + (18) | NRM | + | + |
| 34 | 2, -, CR | Sib, - | MA | - | + | T/MMF | - | - (48) | - | - | - |
| 35 | 1, -, CR | Sib, + | MA | - | + | C/MTX | - | - (20) | - | - | + |
| 36 | 1, -, PR | Sib, - | RI | - | - | C/MMF | - | + (68) | NRM | - | - |
| 37 | 2, -, CR | Sib, + | RI | + | - | C/Prednisone | - | + (25) | NRM | + | + |
| 38 | 2, -, PR | Sib, + | MA | - | + | C/MTX | - | - (57) | - | + | + |
| 39 | 3, -, PD | Sib, + | MA | - | + | C/MTX | - | + (3) | NRM | + | - |
| 40 | 2, -, PD | Sib, - | MA | + | + | C/MTX | + | + (29) | DOD | + | + |
| 41 | 2, -, PR | Sib, - | MA | + | - | C/MMF | - | + (2) | NRM | + | - |
| 42 | 3, -, CR | Sib, + | MA | - | + | C/MTX | - | - (84) | - | - | - |
| 43 | 2, -, CR | Haplo, + | RI | + | + | T/MMF/PTCy | - | + (2) | NRM | - | - |
| 44 | 1, -, PR | MUD, - | MA | + | - | C/MMF/ATG | - | + (20) | NRM | + | + |
| 45 | 2, -, CR | MUD, - | MA | - | + | C/MTX | - | - (34) | - | + | + |
| 46 | 1, -, PR | MUD, - | MA | - | + | C/MMF | - | - (27) | - | - | - |
| 47 | 2, -, PD | MUD, - | MA | + | - | C | - | - (36) | - | - | - |
| 48 | 1, -, PR | MUD, + | RI | + | - | C/MTX | + | - (34) | - | + | - |
| 49 | 1, -, CR | MUD, - | RI | + | - | C/MMF | - | + (11) | NRM | - | - |
| 50 | 2, -, PR | Cord, - | RI | + | - | C/MMF | - | - (53) | - | - | + |
| 51 | 1, -, CR | Sib, - | MA | - | + | T/MMF | - | - (31) | - | + | - |
| 52 | 3, +, PR | Sib, - | RI | + | - | C/MTX | + (4) | + (4) | DOD | + | - |
| 53 | 3, +, CR | MUD, - | RI | - | - | T/MMF | + (8) | - (8) | - | - | - |
| 54 | 3, +, PD | Haplo, - | RI | + | + | T/MMF/PTCy | - | - (13) | - | - | + |

A; acute; ATG: anti-thymocyte globulin; auto-SCT: autologous stem cell transplantation; BM: bone marrow; C: cyclosporine; Ch: chronic; CR: complete remission; DOD: death of disease; Flu: fludarabine; GvHD: acute graft-versus-host disease; Haplo: haploidentical; MA: myeloablative; MMF: mycophenolate mofetil; MTX: methotrexate; MUD: matched unrelated donor; NA: not available; NRM: non-relapse mortality; OS: overall survival; PD: progressive disease; PPx: prophylaxis; PR: partial remission; PTCy: post-transplant cyclophosphamide; RI: reduced-intensity; Sib: matched sibling; T: tacrolimus; TBI: total body irradiation; Tx: therapy

^*^Post-induction

**Table S3: Comparison between the group with RFS > 1.5 years and < 1.5 years**

|  | **RFS > 1.5 years**  **(n = 18)** | **RFS < 1.5 years**  **(n = 26)** | ***P*^1^** |
| --- | --- | --- | --- |
| Continent (n, %)  European  Non-European | 12 (67)  6 (33) | 14 (54)  12 (46) | 0.54 |
| Gender (n, %)  Male  Female | 8 (47)  9 (53) | 22 (85)  4 (15) | 0.02^*^ |
| Age (median, range), years | 36 (8-67) | 36 (12-59) | 0.84 |
| B symptoms (n, %) | 11 (65) | 17 (85) | 0.25 |
| Lymphadenopathy (n, %) | 8 (53) | 7 (28) | 0.18 |
| WBC count (median, range), x10^9^/L | 2.9 (1.6-19) | 6.1 (1.2-80) | 0.45 |
| Hemoglobin (median, range), g/dL | 8 (7.5-13.6) | 9.4 (5.2-14.4) | 0.80 |
| Platelets (median, range), x10^9^/L | 92 (76-263) | 59 (4-121) | 0.07 |
| Subtype (n, %)  γδ αβ | 16 (94)  1 (6) | 17 (77)  5 (23) | 0.21 |
| Immunophenotype (n, %)  CD4^-^CD8^-^  Others | 4 (80)  1 (20) | 13 (87)  2 (13) | 1.00 |
| Disease status at SCT (n, %)  Complete remission  Active disease^2^ | 8 (44)  10 (56) | 9 (38)  15 (62) | 0.76 |
| Number of prior lines of therapy (median, range) | 2 (1-5) | 2 (1-6) | 0.83 |
| Prior auto-SCT (n, %) | 2 (11) | 3 (13) | 1.00 |
| Donor (n, %)  Matched sibling  Others | 10 (59)  7 (41) | 7 (50)  7 (50) | 0.73 |
| Stem cell source (n, %)  Peripheral blood  Bone marrow | 7 (47)  8 (53) | 11 (58)  8 (42) | 0.73 |
| Conditioning intensity (n, %)  Myeloablative  Reduced-intensity | 14 (82)  3 (18) | 10 (59)  7 (41) | 0.26 |
| Acute GvHD (n, %) | 9 (56) | 5 (42) | 0.70 |
| Chronic GvHD (n, %) | 8 (50) | 3 (25) | 0.25 |

Auto-SCT: autologous stem cell transplantation; GvHD: graft-versus-host disease; RFS: relapse-free survival; WBC: white blood cell

^1^Proportions were compared using chi-squared test and Fisher’s exact method when appropriate. Continuous variables were compared using the Mann-Whitney U-test.

^2^Active disease was defined as partial remission or progressive disease.

^*^*P* < 0.05

**References**

1. Voss MH, Lunning MA, Maragulia JC, et al. Intensive induction chemotherapy followed by early high-dose therapy and hematopoietic stem cell transplantation results in improved outcome for patients with hepatosplenic T-cell lymphoma: a single institution experience. *Clinical lymphoma, myeloma & leukemia* 2013; **13**(1): 8-14.

2. Chanan-Khan A, Islam T, Alam A, et al. Long-term survival with allogeneic stem cell transplant and donor lymphocyte infusion following salvage therapy with anti-CD52 monoclonal antibody (Campath) in a patient with alpha/beta hepatosplenic T-cell non-Hodgkin's lymphoma. *Leukemia & lymphoma* 2004; **45**(8): 1673-5.

3. Mansour MR, Dogan A, Morris EC, et al. Allogeneic transplantation for hepatosplenic alphabeta T-cell lymphoma. *Bone marrow transplantation* 2005; **35**(9): 931-4.

4. Machino T, Okoshi Y, Kaneko S, et al. Hepatosplenic alphabeta T-cell lymphoma successfully treated with allogeneic bone marrow transplantation. *Bone marrow transplantation* 2007; **39**(8): 513-4.

5. Basic Kinda S, Durakovic N, Dotlic S, et al. Hepatosplenic alphabeta T-cell lymphoma arising after long-term azathioprine therapy successfully treated with allogeneic bone marrow transplant. *Leukemia & lymphoma* 2013; **54**(6): 1334-5.

6. Lai R, Larratt LM, Etches W, et al. Hepatosplenic T-cell lymphoma of alphabeta lineage in a 16-year-old boy presenting with hemolytic anemia and thrombocytopenia. *The American journal of surgical pathology* 2000; **24**(3): 459-63.

7. Catania G, Zallio F, Monaco F, et al. Successful HLA haploidentical myeloablative stem cell transplantation for aggressive hepatosplenic alpha/beta (alphabeta) T-cell lymphoma. *Leuk Res Rep* 2014; **3**(2): 90-3.

8. Konuma T, Ooi J, Takahashi S, et al. Allogeneic stem cell transplantation for hepatosplenic gammadelta T-cell lymphoma. *Leukemia & lymphoma* 2007; **48**(3): 630-2.

9. Sakai R, Fujisawa S, Fujimaki K, Kanamori H, Ishigatsubo Y. Long-term remission in a patient with hepatosplenic gammadelta T cell lymphoma after cord blood stem cell transplantation following autologous peripheral blood stem cell transplantation. *Bone marrow transplantation* 2006; **37**(5): 537-8.

10. Domm JA, Thompson M, Kuttesch JF, Acra S, Frangoul H. Allogeneic bone marrow transplantation for chemotherapy-refractory hepatosplenic gammadelta T-cell lymphoma: case report and review of the literature. *Journal of pediatric hematology/oncology* 2005; **27**(11): 607-10.

11. Aldinucci D, Poletto D, Zagonel V, et al. In vitro and in vivo effects of 2'-deoxycoformycin (Pentostatin) on tumour cells from human gammadelta+ T-cell malignancies. *British journal of haematology* 2000; **110**(1): 188-96.

12. Belhadj K, Reyes F, Farcet JP, et al. Hepatosplenic gammadelta T-cell lymphoma is a rare clinicopathologic entity with poor outcome: report on a series of 21 patients. *Blood* 2003; **102**(13): 4261-9.

13. Cooke CB, Krenacs L, Stetler-Stevenson M, et al. Hepatosplenic T-cell lymphoma: a distinct clinicopathologic entity of cytotoxic gamma delta T-cell origin. *Blood* 1996; **88**(11): 4265-74.

14. Gassas A, Kirby M, Weitzman S, Ngan B, Abla O, Doyle JJ. Hepatosplenic gammadelta T-cell lymphoma in a 10-year-old boy successfully treated with hematopoietic stem cell transplantation. *American journal of hematology* 2004; **75**(2): 113-4.

15. He S, Roberts A, Ritchie D, Grigg A. Graft-versus-lymphoma effect in progressive hepatosplenic gamma/delta T-cell lymphoma. *Leukemia & lymphoma* 2007; **48**(7): 1448-50.

16. Przybylski GK, Wu H, Macon WR, et al. Hepatosplenic and subcutaneous panniculitis-like gamma/delta T cell lymphomas are derived from different Vdelta subsets of gamma/delta T lymphocytes. *The Journal of molecular diagnostics : JMD* 2000; **2**(1): 11-9.

17. Rossbach HC, Chamizo W, Dumont DP, Barbosa JL, Sutcliffe MJ. Hepatosplenic gamma/delta T-cell lymphoma with isochromosome 7q, translocation t(7;21), and tetrasomy 8 in a 9-year-old girl. *Journal of pediatric hematology/oncology* 2002; **24**(2): 154-7.

18. Weidmann E, Hinz T, Klein S, et al. Cytotoxic hepatosplenic gammadelta T-cell lymphoma following acute myeloid leukemia bearing two distinct gamma chains of the T-cell receptor. Biologic and clinical features. *Haematologica* 2000; **85**(10): 1024-31.

19. Mittal S, Milner BJ, Johnston PW, Culligan DJ. A case of hepatosplenic gamma-delta T-cell lymphoma with a transient response to fludarabine and alemtuzumab. *European journal of haematology* 2006; **76**(6): 531-4.

20. Falchook GS, Vega F, Dang NH, et al. Hepatosplenic gamma-delta T-cell lymphoma: clinicopathological features and treatment. *Annals of oncology : official journal of the European Society for Medical Oncology / ESMO* 2009; **20**(6): 1080-5.

21. Jonveaux P, Daniel MT, Martel V, Maarek O, Berger R. Isochromosome 7q and trisomy 8 are consistent primary, non-random chromosomal abnormalities associated with hepatosplenic T gamma/delta lymphoma. *Leukemia* 1996; **10**(9): 1453-5.

22. Takaku T, Miyazawa K, Sashida G, et al. Hepatosplenic alphabeta T-cell lymphoma with myelodysplastic syndrome. *International journal of hematology* 2005; **82**(2): 143-7.

23. Tanase A, Schmitz N, Stein H, et al. Allogeneic and autologous stem cell transplantation for hepatosplenic T-cell lymphoma: a retrospective study of the EBMT Lymphoma Working Party. *Leukemia* 2015; **29**(3): 686-8.
